# Phytostabilization of Pb-Zn Mine Tailings with *Amorpha fruticosa* Aided by Organic Amendments and Triple Superphosphate

**DOI:** 10.3390/molecules25071617

**Published:** 2020-04-01

**Authors:** Ashim Sikdar, Jinxin Wang, Mirza Hasanuzzaman, Xiaoyang Liu, Shulin Feng, Rana Roy, Tanveer Ali Sial, Altaf Hussain Lahori, Parimala Gnana Soundari Arockiam Jeyasundar, Xiuqing Wang

**Affiliations:** 1 College of Natural Resources and Environment, Northwest A&F University, Yangling 712100, China; ashim.aes@sau.ac.bd (A.S.); ranaroy.aes@sau.ac.bd (R.R.); or alisial@nwafu.edu.cn (T.A.S.); apgs_1987@yahoo.co.in (P.G.S.A.J.); 2 Department of Agroforestry and Environmental Science, Sylhet Agricultural University, Sylhet 3100, Bangladesh; 3 Key Laboratory of Plant Nutrition and the Agri-Environment in Northwest China, Ministry of Agriculture, Yangling 712100, China; 4 Department of Agronomy, Faculty of Agriculture, Sher-e-Bangla Agricultural University, Dhaka 1207, Bangladesh; mhzsauag@yahoo.com; 5 Institute of Soil and Water Conservation, Northwest A&F University, Yangling 712100, China; liuxiaoyang_hazel@outlook.com (X.L.); shulin_feng@yahoo.com (S.F.); wangxiuqing_work@163.com (X.W.); 6 Department of Soil Science, Sindh Agriculture University, Tandojam 70060, Pakistan; 7 Department of Environmental Sciences, Sindh Madressatul Islam University, Karachi 74000, Pakistan; ahlahori@yahoo.com

**Keywords:** heavy metal stabilization, organic amendments, triple superphosphate, *Amorpha fruticosa*, phytoremediation efficiency, enzymatic antioxidants

## Abstract

A greenhouse pot trial was conducted to investigate the effect of organic amendments combined with triple superphosphate on the bioavailability of heavy metals (HMs), *Amorpha fruticosa* growth and metal uptake from Pb-Zn mine tailings. Cattle manure compost (CMC), spent mushroom compost (SMC) and agricultural field soil (AFS) were applied to tailings at 5%, 10%, 20% and 30% *w*/*w* ratio, whereas sewage sludge (SS) and wood biochar (WB) were mixed at 2.5%, 5%, 10% and 20% *w*/*w* ratio. Triple superphosphate (TSP) was added to all the treatments at 4:1 (molar ratio). Amendments efficiently decreased DTPA-extracted Pb, Zn, Cd and Cu in treatments. Chlorophyll contents and shoot and root dry biomass significantly (*p* < 0.05) increased in the treatments of CMC (except T4 for chlorophyll b) and SMC, whereas treatments of SS (except T1 for chlorophyll a and b), WB and AFS (except T4 for chlorophyll a and b) did not show positive effects as compared to CK1. Bioconcentration factor (BCF) and translocation factor (TF) values in plant tissues were below 1 for most treatments. In amended treatments, soluble protein content increased, phenylalanine ammonialyase (PAL) and polyphenol oxidase (PPO) decreased, and catalase (CAT) activity showed varied results as compared to CK1 and CK2. Results suggested that *A. fruticosa* can be a potential metal phytostabilizer and use of CMC or SMC in combination with TSP are more effective than other combinations for the in situ stabilization of Pb-Zn mine tailings.

## 1. Introduction

Mine tailings or spoils are residues generated due to mineral grinding after recovering precious metals by conventional mining processes that are major sources of metallic contamination worldwide. These tailings are characterized by potentially toxic concentrations of heavy metals (HMs), poor physical properties, high wind and water erosion susceptibility, and eventually pose serious risks to the environment and human health [1,2]. In China, there are over 8000 national mining enterprises and 230,000 private mines, from which hundreds of millions of tons of mining wastes are accumulated annually [3]. Because of the production of such a huge amount of mining residues, China is facing serious environmental problems [1,2,3], therefore, the development of economical and efficient technologies for remediation of mine tailings wastelands is an urgent issue of concern, not only in China but globally.

The conventional technologies for rehabilitating metal contaminated sites such as incineration, landfilling or soil washing, solidification/stabilization, electrokinetic treatment and soil excavation have been reported to be impractical, especially for large tailings areas due to high cost, high energy consumption, complex protocols and possible secondary pollution [4,5]. In contrast, phytoremediation represents a cost-effective and environmentally sustainable reclamation technique for the removal or stabilization of mine soil HMs using plants that has emerged during the last decades. Among the various phytoremediation techniques, phytostabilization, i.e., the use of metal-tolerant plants for sequestration of the metals within the roots and rhizosphere [6], is a relevant remediation strategy for metal contaminated mine tailings that facilitates the establishment of a vegetation cover for the long-term stabilization and reclamation of tailings. Since mine tailings represent a very unfavorable environment for plant growth [4], suitable amendments must be added to stabilize the metals in situ as well as to improve their physicochemical status to make these sites more favorable for plant growth and survival. When such an attempt at vegetation is conducted combined with metal immobilizing agents, i.e., amendments, it is often termed “assisted or aided phytostabilization” [7,8,9].

Although a large number of organic and inorganic amendments have been used to assist and reinforce phytostabilization processes so far, industrial and farm wastes or byproducts attract special interest because of their availability, low price and importantly positive contribution to the circular economy through recycling. In addition, the use of a single amendment may not be appropriate for immobilizing all metals of concern due to the diversity of the bonding behavior between the soil matrix and HMs [10]. Organic wastes such as manure compost and sewage sludge have been proposed as cost-effective and efficient soil amendments as they can improve soil health and fertility by increasing organic matter content, nutrient levels and water retention capacity [4,11,12]. Spent mushroom compost that is abundantly generated by the mushroom growing industries, can be applied for the stabilization of mine sites as it is a slow release fertilizer and it has vast sorption capacity for lead, cadmium and chromium due to the presence of phosphoryl, hydroxyl and phenolic functional groups on its surface [13,14]. Biochar is a product of organic material pyrolysis that has emerged as one of the most promising additives to remediate degraded soils due to its HM adsorption capacity, high levels of recalcitrant organic carbon and also the ability to alter soil microbial abundance [15]. Incorporation of topsoil from an unmined site onto mine tailings is a commonly practiced technique for reducing the concentrations of HMs [16]. On the other hand, among inorganic amendments, the use of phosphate fertilizers has been shown to be potentially efficient as an in situ remediation technique for metal-contaminated mine wastes [10,17]. Phosphate fertilizers can effectively stabilize soil Pb by the formation of lead phosphates which are the most insoluble and stable forms of Pb in soils. Although P fertilizers have generally been used to remediate Pb-contaminated sites, they may also be effective on other metals such as Cd, Zn and Cu [18]. A 4:1 P/Pb molar ratio was prescribed to be sufficient and environmentally safe (devoid of eutrophication possibility) for sites contaminated with elevated Pb concentrations [19,20].

The success of the phytostabilization process depends on the selection of suitable plant species. Previous studies have shown that plant species with deep root systems, high biomass production capacity, tolerance to elevated metal concentrations, low translocation of metals from roots to aerial parts and adaptation ability for poor soil nutrients are appropriate for phytostabilization [21]. Therefore, *A. fruticosa* (false indigo), a deciduous shrub on the Fabaceae family was chosen as study plant in this work because of its tap root system, nitrogen fixing ability, capacity to grow in adverse conditions and ability to re-sprout from the stumps after harvesting [22]. It is native to Canada, Mexico and United States, but has become naturalized in many parts of Asia, including China [23].

Abiotic stresses including metal stress cause impairment in the photosynthetic system and disturbance in physiological processes, leading to stunted plant growth and poor biomass yield [24,25]. The imbalance in physiological activities occurs mainly because of the generation of excess reactive oxygen species (ROS) induced by toxic metals [26,27]. In order to scavenge ROS and avoid oxidative stress, plants produce and activate different antioxidant enzymes like catalase (CAT), phenylalanine ammonialyase (PAL) and polyphenol oxidase (PPO) as a defense mechanism [28]. Like antioxidant enzymes, soluble proteins also play an important role in plant defense system by regulating the osmotic balance, stabilizing macromolecules, quenching ROS and preventing oxidative damage, and thereby are considered as bioindicators of metal stress [29,30,31]. Hence, it is important to evaluate the physiological defensive capacity of plants under metal stress for successful phytoremediation processes.

Several researchers have studied the effect of different types of amendments in assisted phytostabilization of tailings [7,11,12,16,32,33]. Here, we evaluated the effects of some organic amendments such as cattle manure compost (CMC), spent mushroom compost (SMC), sewage sludge (SS), wood biochar (WB) and agricultural field soil (AFS), each combined with triple superphosphate (TSP, P/Pb molar ratio 4:1) on the immobilization and bioavailability of HMs (Pb, Zn Cd and Cu) in Pb-Zn mine tailings. We selected TSP with the established optimum application rate for metal immobilization and organic additives to stabilize metals as well as to improve soil health for supporting plant growth, which are the two important issues that should be focused on. In addition, we attempted direct seed sowing in lieu of transplanting, which is rare especially in case of tailings phytostabilization. In our current study, we hypothesized that: (1) TSP combined with CMC, SMC, SS, WB and AFS may improve tailings properties, reduce metal bioavailability and thereby promote plant growth and decrease metal uptake by *A. fruticosa* and (2) *A. fruticosa* may activate its’ antioxidant defense system and show its’ potential as a phytostabilizer for the studied site. Therefore, the specific objectives of this study were: (1) to determine the effect of combined amendments (organic amendments plus TSP) application on tailings pH, electrical conductivity (EC) and the bioavailability of HMs (Pb, Zn Cd and Cu); (2) to investigate the growth response, metal uptake, antioxidant enzyme activities (CAT, PAL and PPO) and soluble protein content of *A. fruticosa* after amending the tailings jointly with organic amendments and TSP.

## 2. Results and Discussion

### 2.1. Effect of Amendments on pH and EC of Tailings

Soil pH and EC are important factors that directly influence metal bioavailability just as soil acidity and salinity determine the solubility and mobility of metals in soil solution [34,35]. In the present study, pH and EC were significantly (*p* < 0.05) affected by the amendments over the CK1 and CK2. The pH of CK1 and CK2 were 6.64 and 6.63, respectively, whereas in amended samples the pH ranged from 6.25 to 7.35 (Table 1). The maximum pH (7.35) was recorded in WBT4 and the minimum (6.25) in SST4. This result is due to the fact that biochar has a liming effect that contributes in increasing soil pH [36,37]. The incorporation of SS decreased soil pH owing to the release of organic acids during organic matter decomposition by microbes [38,39].

Soil EC increased with increasing application rates of amendments in their respective treatments ranging from 1.08 to 4.65 dS m^−1^. There was no significant difference in EC values between CK1 and CK2 (Table 1). The highest EC range was observed in WB (1.61 to 4.65 dS m^−1^), followed by SS (1.24 to 2.31 dS m^−1^), CMC (1.24 to 1.79 dS m^−1^), SMS (1.08 to 1.41 dS m^−1^) and AFS (1.18 to 1.20 dS m^−1^). Our results regarding soil EC are consistent with the findings of previous studies [32,39,40,41]. WB greatly increased soil EC with the increasing application rates as compared to other organic amendments might be due to its’ high ash content as our studied biochar was produced from apple wood, and similar results were reported by Lomaglio et al. [42] and Sial et al. [36,37].

### 2.2. DTPA-Extracted Pb, Zn, Cd and Cu Concentrations in Treated Tailings

**Figure 1:**
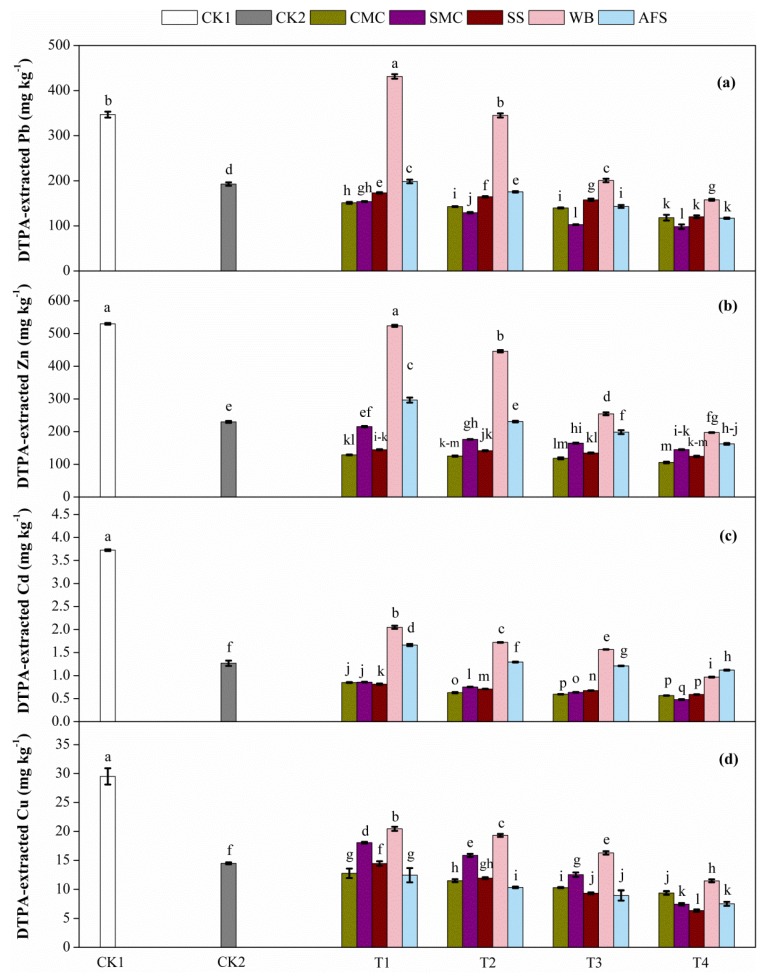
Effect of amendments on DTPA-extractable Pb (**a**), Zn (**b**), Cd (**c**) and Cu (**d**) in treated tailings. CK1= Only tailings and CK2= Tailings plus TSP. The amendment treatments are denoted as T1, T2, T3 and T4. Error bars represent the standard deviation of the mean (*n* = 3). Letters above the bars refer to the difference at significance level (*p* < 0.05, LSD) among different treatments. CK1: Control 1; CK2: Control 2; CMC: Cattle manure compost; SMC: Spent mushroom compost; SS: Sewage sludge; WB: Wood biochar; AFS: Agricultural field soil; DTPA: Diethylenetriaminepentaacetic acid (for interpretation of the references to color in this figure legend, the reader is referred to the web version of this article).

After 180 days of pot experiments, the effect of amendments on the bioavailability of Pb, Zn, Cd and Cu was determined using the DTPA extraction method and the results shown in Figure 1. A significant reduction was reported in the tailing bioavailable metal concentrations (Pb, Zn, Cd and Cu) after receiving the different amendments except for WB-treated samples with respect to both CK1 and CK2. DTPA Pb content between CK1 and CK2 was significantly (*p* < 0.05) different. The bioavailable Pb contents decreased by 56.4–65.9% in CMC, 55.6–71.7% in SMC, 50.1–65.3% in SS and 42.8–66.2% in AFS treated tailings than CK1, and by 21.6–38.7% in CMC, 20.2–49.1% in SMC, 10.2–37.6% in SS than CK2. In contrast, DTPA Pb content increased only in WBT1 than CK1, and in WBT1, WBT2, WBT3 and AFST1 more than CK2. The highest DTPA Pb content was determined in WBT1 (431.13 mg kg^−1^) followed by CK1 (346.61 mg kg^−1^) and the lowest value was reported in SMCT4 (98.15 mg kg^−1^) followed by SMCT3 (102.75 mg kg^−1^). Pb was better immobilized by SMC and CMC followed by AFS, SS and WB (Figure 1a). In addition, our results indicated that Pb was more efficiently immobilized in most treatments with combined amendments than the treatment with TSP alone (CK2). Considering the factors affecting Pb immobilization by phosphate compounds, we hypothesize that, the reduction in bioavailability of Pb could be attributed to dissolution of P or meta-stable Pb compounds and subsequent precipitation of stable minerals like pyromorphite that might have occurred due to the presence of Pb and P provided by tailings and TSP, respectively. Moreover, the immobilization of Pb might have occurred also through sorption reactions along with Pb carbonate and Pb sulphate formation as in our treated tailings pH (6.25–7.35) were not low enough and similar interpretations was reported by Zhang et al. [43]. We also assume that organic matter added from the organic amendments efficiently contributed to Pb stabilization [44].

Like Pb, Zn was also effectively immobilized in tailings after mixing with the different amendments. CK2 had significantly (*p* < 0.05) lower bioavailable Zn content than CK1. The decreasing trends of Zn contents were 75.7–80.1% in CMC, 59.4–72.7% in SMC, 72.7–76.6% in SS, 1.2–62.8% in WB and 44–69.4% in AFS treated samples as compared to CK1, whereas in comparison to CK2, the decreasing trends were 43.9–54% in CMC, 6.3–36.9% in SMC, 37.1–45.9% in SS treated samples. WB and AFS treated samples also showed a decreasing trend in bioavailable Zn content, but Zn concentrations increased in the treatments with lower doses of WB and AFS as compared to CK2 (Figure 1b). Therefore, all the organic amendments used combined with TSP were capable in reducing the bioavailability of Zn in Pb-Zn mine tailings. Apart from Pb, mechanisms of Zn, Cd and Cu immobilization with P compounds like TSP might be co-precipitation and surface complexation, resulting in the formation of compounds which are less stable than minerals like pyromorphite [45]. In our study, pH in treated samples did not significantly alter; therefore, increase in soil cation exchange capacity (CEC, data not shown) might be the possible reason for the diminution of Zn solubility, and this result is in agreement with Huang et al. [46]. In addition, sufficiently humified organic matter provided by organic amendments might have contributed a lot to decrease Zn mobility through stable chelate formation [47].

In case of Cd immobilization, a highly significant difference was observed between CK1 and CK2, and amendments also showed significant (*p* < 0.05) differences in maximum treatments as compared to CK1 and CK2. The Cd contents were reduced in the range of 77.2–84.8% in CMC, 76.9–87.2% in SMC, 78.2–84.2% in SS, 44.9–74% in WB and 55.3–69.9% in AFS in comparison to CK1. On the other hand, the decreasing trends of Cd contents were 33.1–55.4% in CMC, 32.5–62.4% in SMC and 36–53.6% in SS treated samples as compared to CK2. A decreasing trend in bioavailable Cd content was also reported in WB- and AFS-treated samples with increasing application rates; but Cd contents were higher in tailings treated with WB by 61.4–23.5% (except WBT4 where decreased by 23.9%) than CK2 while in AFS, treatments with low doses (5% and 10%) had higher Cd contents and treatments with high doses (20% and 30%) showed lower Cd contents than CK2 (Figure 1c). The highest DTPA Cd content was reported in CK1 (3.72 mg kg^−1^) followed by WBT1 (2.05 mg kg^−1^) and the lowest value was determined in SMCT4 (0.48 mg kg^−1^) followed by CMCT4 (0.57 mg kg^−1^). As phosphate compounds are normally more effective in forming stable phosphate minerals with Pb than with other metals, the decrease in Cd content in our studied tailings might be ascribed to the absorption mechanisms like surface complexation and/or ion exchange instead of formation of cadmium phosphate [48]. Valipour et al. [49] reported that TSP significantly reduced DTPA-extractable Cd content by 10–29% in contaminated soils from four provinces in Iran. Besides, in this study, organic matter provided by the organic amendments might have played an efficient role in Cd immobilization through adsorption reactions. Chen et al. [50] observed that in contaminated alfisol 48–70% transformation of soluble Cd to organic bond Cd occurred by compost application. Although biochar is well known for its HMs adsorption capacity, WB treated tailings showed higher bioavailable Cd content as compared to other amendments, and this result might be because of the fact that biochar is comparatively more recalcitrant and resilient to degradation, and eventually it needs longer time to mineralize through physical or biological processes than other organic amendments [51].

Addition of amendments to tailings resulted in significant differences in DTPA extractable Cu content as compared to control treatments. CK1 and CK2 showed a highly significant (*p* < 0.05) difference in bioavailable Cu content. The decreasing trends of Cu contents were 56.8–68.2% in CMC, 38.8–74.7% in SMC, 51–78.4% in SS, 30.7–61.2% in WB and 57.9–74.5% in AFS treated samples as compared to CK1, whereas in comparison to CK2, the decreasing trends were shown in CMC, SS and AFS treated samples in the range of 11.9–35.1%, 0.2–56.1% and 14.2–48.1%, respectively. DTPA Cu content increased in treatments with lower SMC doses (5% and 10%) and attenuated in higher SMC doses (20% and 30%) than CK2. Like SMC treatments, Cu content in WB-treated samples also decreased with the increase in WB application rates; but compared to CK2, Cu content increased in T1, T2 and T3 whereas in T4 (20% WB) reduced Cu content was reported (Figure 1d). Compared to CK1 and CK2, the maximum efficacy in Cu immobilization was shown by SS treatments up to 78.4% and 56.1%, respectively, followed by SMC, AFS, CMC and WB treatments. pH and organic matter are the important factors in regulating Cu mobility in soil. Since pH range (6.25–7.35) in our treatments was near neutral, it could not contribute much in controlling Cu mobility. Moreover, P amendment was less efficient for Cu immobilization than for Pb, as previously mentioned. Therefore, organic matter might have played a fundamental role in controlling bioavailable Cu concentration. Lombi, et al. [52] and Mehlhorn et al. [53] reported that Cu has a high affinity for organic matter and eventually it can be immobilized either by firm attachment to dissolved organic matter or sequestered by adsorption to solid-phase organic matter. Our results are consistent with the findings of Branzini and Zubillaga [10], who revealed that soluble and exchangeable Cu fractions reduced in contaminated soil treated with biosolid compost due to the mechanism of precipitation and complexation by humic substances present in organic matter. Meier, et al. [54] also reported a considerable decrease in exchangeable Cu fraction in soil treated with chicken manure-made biochar.

### 2.3. Impact of Amendments on Dry Biomass Yield and Chlorophyll Content

The results in terms of dry biomass yield and chlorophyll contents (a and b) of *A. fruticosa* have been presented in Table 1. The application of CMC and SMC significantly improved plant dry biomass yield, but AFS, WB and SS amendments had no significant effect as compared to both CK1 and CK2. The highest shoot and root biomass (13.48 g pot^−1^ and 19.45 g pot^−1^) were obtained for CMCT3 (20% CMC) and the lowest for SST4 (1.75 g pot^−1^ and 0.71 g pot^−1^). In case of CMC and SMC, the highest application rate (30%) reduced shoot and root biomass as compared to 5, 10 and 20% rates. However, WB showed a decreasing trend in shoot and root biomass yield with increasing rate of biochar, and no plants had survived after few days of germination in WBT4. Our findings coincide with previous studies where addition of SMC [32] and CMC [55] improved plant dry biomass yield in Pb-Zn mine tailings. Solís-Dominguez et al. [11] also observed pronounced biomass yield in some woody species (*Prosopis juliflora*, *Acacia greggi*, *Cercocarpus montanus* and *Atriplex lentiformis*) with 15% and 20% compost application into mine tailings, which afterwards was effectively scaled to the field by Gil-Loaiza et al. [33]. The maximum dose of CMC and SMC (30%) resulted in reduced biomass yield might be because of the elevated activity of higher microbial population and slow degradation rate under sandy environment of studied tailings [38]. Chiu et al. [40] suggested that higher dose of SS (>10%) could not increase biomass production and similar result was observed in our study, i.e., the lowest plant biomass yielded under highest rate of SS (20%). In addition, plants did not grow in WBT4 because of elevated EC (4.65 dS m^−1^) [56]. Besides, biochar application at higher rates may promote metal immobilization, but may also result in harmful effects like reduction in micronutrients, decrease in CEC, excessive increase in EC and development of some phytotoxic symptoms that may eventually hinder biomass yield [57]. However, the addition of AFS to mine tailings had no significant effect on shoot and root dry biomass and this result are in line with Zhang et al. [16].

**Figure 2:**
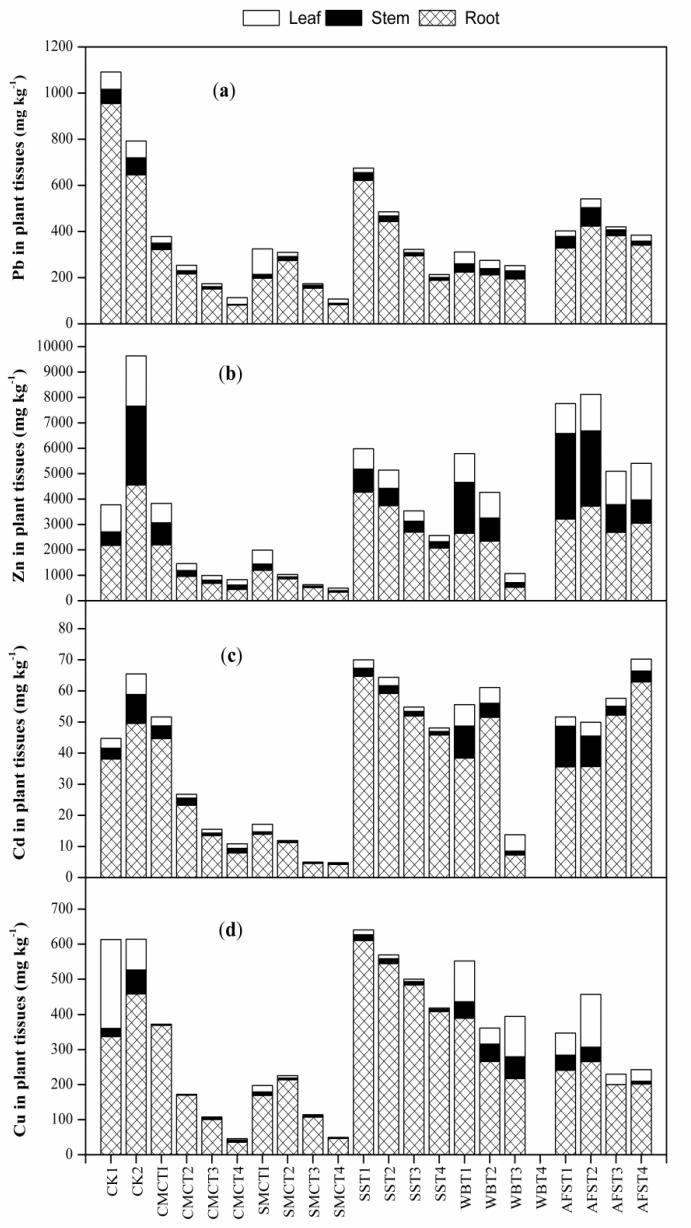
Effect of amendments on concentrations of Pb (**a**), Zn (**b**), Cd (**c**) and Cu (**d**) in *A. fruticosa* tissues grown in treated tailings. Letters above the bars refer to the difference at significance level (*p* < 0.05, LSD) among different treatments. CK1: Control 1; CK2: Control 2; CMC: Cattle manure compost; SMC: Spent mushroom compost; SS: Sewage sludge; WB: Wood biochar; AFS: Agricultural field soil. Plant did not grow in WBT4.

HMs can hinder chlorophyll biosynthesis in plants through inhibition of necessary enzyme activities or by promoting reactive oxygen species (ROS) generation [58]. Incorporation of amendments to tailings exerted significant (*p* < 0.05) effect on chlorophyll a, and chlorophyll b content as compared to both CK1 and CK2 (Table 1). Notably, chlorophyll contents were significantly (*p* < 0.05) higher in SMC treated samples than CK1 and CK2. The highest chlorophyll a (3.29 mg g^−1^FW) and chlorophyll b (1.17 mg g^−1^FW) contents were reported in SMCT3, which were 93.4% and 71.9% higher than CK1 and 235.4% and 215.9% higher than CK2, respectively. In contrast, the lowest chlorophyll a (0.10 mg g^−1^FW) and chlorophyll b (0.10 mg g^−1^FW) contents were determined in WBT3, which was 94.2% and 85.7% lower than CK1 and 89.9% and 73.7% lower than CK2, respectively. During pot experiment, chlorotic spots were observed on *A. fruticosa* leaves, possibly due to the higher total Zn concentration (15,439 mg kg^−1^) in tailings. The higher concentration of Zn may replace Mg, which promotes the degradation of photosystems as less stable Zn-chlorophyll, thus developing leaf chlorosis [59]. Cd was also reported to impair chlorophyll production in plant leaves [60]. In our study, SMC and CMC application increased leaf chlorophyll contents, which is similar to the results of Wang et al. [55]. Poor chlorophyll contents in SS, WB and AFS treated samples might be due to low uptake of Mg and Fe by plants that are directly related to chlorophyll synthesis [61]. Moreover, leaf Cu concentrations in WB and AFS treatments were above 20−30 mg kg^−1^ (Figure 2d), which might have resulted in direct damage of PSII, thereby produced ROS that eventually retarded photosynthesis [59]. Chai et al. [62] also determined reduced leaf chlorophyll content in *Spartina alterniflora* due to HMs.

### 2.4. Effect of Amendments on Pb, Zn, Cd and Cu Uptake by A. Fruticosa

The average concentrations of Pb, Zn, Cd and Cu uptake in different parts of *A. fruticosa* plants grown in amended and un-amended tailings have been summarized in Figure 2. Bonanno [63] stated that plant type, the total and bioavailable metal concentration in soil are the main factors that regulate the metal uptake in plants. In this study, the Pb concentrations varied widely from 80.93–954 mg kg^−1^ in root, 3.45–80.26 mg kg^−1^ in stem and, 6.71–110 mg kg^−1^ in leaf, taking all the treatments into account. Pb concentrations in plant tissues were significantly (*p* < 0.05) different between CK1 and CK2. The Pb uptake in root was significantly reduced in all the treated samples than both CK1 and CK2 (Figure 2a). The highest root Pb concentration was found in CK1 (954 mg kg^−1^) followed by CK2 (645 mg kg^−1^) whereas the lowest root Pb was reported in CMCT4 (80.93 mg kg^−1^) followed by SMCT4 (82.94 mg kg^−1^). Comparatively, concentrations of root Pb were lower in CMC and SMC treated samples than that of the samples treated with other amendments. We assume that in CMC and SMC treated samples, Pb not only stabilized as insoluble Pb compounds in root surface [64] and in tailings [65], but also immobilized through the fixation by organic matter under favorable pH and EC [66]. In contrast, the increased Pb uptake by *A. fruticosa* roots in SS, WB and AFS treated tailings might be explained by their higher bioavailable Pb concentrations due to high salinity. The treatment having the highest salinity (WBT4) did not support plant growth. Compared to CK1 and CK2, stem Pb concentrations in all the treatments had significant difference. Like root Pb, CMC and SMC treatments were reported to have lower stem Pb concentration as compared to the treatments with other amendments. The highest and lowest stem Pb concentration was recorded in AFST2 (80.26 mg kg^−1^) and CMCT4 (3.45 mg kg^−1^), respectively. In case of leaf Pb, significant differences also found in all the treated samples as compared to control treatments. Though unexpectedly the highest leaf Pb concentration was determined in SMCT1 (110.57 mg kg^−1^), it was reduced to the lowest in SMCT3 (6.71 mg kg^−1^). However, it is evident that higher Pb concentrations were determined in roots as compared to other tissues. This phenomenon is consistent with the fact that Pb is comparatively less mobile in plant tissues and eventually translocation of Pb from root to aerial parts is limited [65]. In terms of the phytotoxic range of Pb in plants (30−300 mg kg^−1^), many treatments were found to be in phytotoxic ranges [67]. Nevertheless, considering the initial total Pb concentration of studied tailings (5614 mg kg^−1^), the reasonable Pb uptake in root and subsequent limited translocation to aerial parts suggests that *A. fruticosa* is suitable for phytostabilization of mine tailings.

The concentrations of Zn in *A. fruticosa* tissues were much higher than those of Pb, Cd and Cu, ranging from 335.88–4555 mg kg^−1^ in root, 58.58–3372 mg kg^−1^ in stem and, 53.58–1978 mg kg^−1^ in leaf. This might be because of the elevated Zn concentration (15,439 mg kg^−1^) in studied tailings. According to Kabata-Pendias [67], the phytotoxic level of Zn in plants was defined to be 100–400 mg kg^−1^ and considering this limit, Zn concentration in plant tissues not only reached the phytotoxic limit in most treatments but also exceeded the range in many cases.CK1 and CK2 were statistically significant (*p* < 0.05) regarding Zn concentrations in all plant tissues. In case of root Zn, a significant reduction was noticed in all the treatments as compared to CK2 while in some treatments especially SS, WB and AFS treated samples, root Zn concentrations were higher than CK1 (Figure 2b). However, the highest and lowest root Zn concentrations were observed in CK2 (4555 mg kg^−1^) and SMCT4 (335 mg kg^−1^), respectively. Contrary to root Zn, AFST1 was reported to have the highest Zn concentration in stem (3372 mg kg^−1^) whereas the lowest value was determined in SMCT3 (58.58 mg kg^−1^). Leaf Zn concentration showed the same trend as root Zn as compared to CK1 and CK2. The minimum leaf Zn concentration was determined in SMCT3 (53.58 mg kg^−1^) and the maximum Zn translocation to leaf occurred in CK2 (1978 mg kg^−1^). In all cases of root, stem and leaf Zn concentrations, every treatment was statistically different as compared to CK1 and CK2. Like Pb, plants grown in SS, WB and AFS treated tailings retained more Zn in root than in treatments with CMC and SMC. In the present study, the translocation rate of Zn from root to aerial portion was evidently higher in most treatments in comparison to Pb which indicates that different metals have different mobility patterns within plants. Pulford and Watson [21] revealed that Cd and Pb tended to be stabilized and retained in roots, whereas Zn and Cu normally translocated to shoots. In addition, Pb and Cd are known to be non−essential metals and toxic to plants in higher amount [68], while Zn and Cu are essential elements for plant growth and play a significant role in protein synthesis and photosynthesis [69]. Therefore, plants naturally develop their mechanism to prevail against metal stress by translocating necessary elements (Zn and Cu) to shoots and holding more harmful metals (Pb and Cd) in roots to protect the aerial parts from toxic effects. In this context, our results are in accordance with Yang et al. [70].

Cd concentration in *A. fruticosa* tissues was reported to be the lowest among the studied HMs, ranging from 4.21−64.65 mg kg^−1^ in root, 0.29−13.10 mg kg^−1^ in stem and, 0.16−6.92 mg kg^−1^ in leaf. Regarding Cd uptake, Pandey [71] reported that Zn competes with Cd as the transportation of both metals is conducted by a common carrier at root plasma membrane, and thus Zn restricts Cd uptake by plants. Moreover, assimilation of Zn by plants is easier than Cd; hence, Cd accumulation reduces in presence of Zn [72]. However, the concentrations of Cd in the above-ground parts in most treatments were below phytotoxic level established for plants (5−30 mg kg^−1^) [67]. In our study, a significant difference was shown in Cd uptake in root CK1 and CK2. Besides, all the treatments were found statistically significant (*p* < 0.05) as compared to both CK1 and CK2 (Figure 2c). Compared to CK1, root Cd concentration decreased in the range of 39−79.3% in CMC except T1 (17.2% increased), 63.5−89% in SMC, 6.5−6.7% in AFS treated samples except T3 and T4 (37.1−64.9% increased), whereas increased from 20.2−69.7% in SS and 0.8−35.1% in WB treated samples except T3 (80.9% decreased). Contrarily, the root Cd significantly decreased from 9.9−84% in CMC, 71.9−91.5% in SMC, 22.5−85.3% in WB except for T2 (3.9% increased) and 28.1−28.2% in AFS treated samples except for T3 and T4 (5.4−26.8% increased), while increased from 4.8−30.5% in SS except T4 (7.6% decreased) when compared to CK2. Therefore, CMC and SMC addition reduced the Cd uptake in root, with the minimum being in SMCT4 (4.21 mg kg^−1^); while other amendments showed a varying trend. The reduction in root Cd in CMC and SMC might be occurred by Cd−complex formation with organic matter which reduced the phytoavailability of Cd to *A. fruticosa*. Mahar et al. [44] also reported the same findings. Furthermore, in CMC and SMC treated tailings, organic matter from compost and P addition from TSP jointly resulted in higher biomass production under suitable pH and EC which could offset the decrease in Cd uptake [17]. The lowest and highest Cd concentration in stem was reported in SMCT3 (0.29 mg kg^−1^) and AFST1 (13.10 mg kg^−1^), whereas SMCT3 (0.16 mg kg^−1^) and WBT1 (6.92 mg kg^−1^) were noted to have the lowest and highest value of leaf Cd concentration. Like Pb, Cd translocation from root to aerial parts was relatively lower than Zn and Cu.

Cu uptake and accumulation in *A. fruticosa* tissues were much lower than Pb and Zn, ranging from 35.69−609 mg kg^−1^ in root, 0.13−67.74 mg kg^−1^ in stem and 0.97−253 mg kg^−1^ in leaf. Cu uptake in root was significantly higher in CK2 than CK1. The concentration of root Cu ranged from 35.69−369 mg kg^−1^ in CMC, 46.18−169 mg kg^−1^ in SMC, 408−609 mg kg^−1^ in SS, 217−389 mg kg^−1^ in WB and 199−265 mg kg^−1^ in AFS treated samples, with the highest being in SST1 (609 mg kg^−1^) and the lowest being in CMCT4 (35.69 mg kg^−1^). Root Cu concentration in CMC (except T1), SMC, WB (except T1) and AFS treatments considerably reduced than CK1, whereas in SS treatments significantly increased as compared to CK1 (only tailings) value (Figure 2d). In contrast, root Cu in only SS treatments (except T4) exceeded CK2 (only with TSP) value. In agreement with our findings, Seo, et al. [22] also reported lower root Cu concentration in mixed fertilizer treated samples (131 mg kg^−1^) than organic fertilizer treated (226 mg kg^−1^), inorganic fertilizer treated (223 mg kg^−1^) and unfertilized samples (215.7 mg kg^−1^). In our investigation, SS treated samples showed increased root Cu concentration than CK1 and CK2 might be due to greatly higher Cu concentration in SS (51.46 mg kg^−1^) relative to other amendments. CMC and SMC treatments showed lower Cu uptake in roots than SS, WB and AFS treatments, and eventually Cu translocation to stem and leaf was also in smaller amount than other treatments. Like Zn, Cu uptake in root and translocation to shoots were higher than Pb and Cd in amended and unamended tailings. The maximum and minimum Cu concentration in stem were determined in CK2 (67.74 mg kg^−1^) and AFST3 (0.13 mg kg^−1^), whereas CK1 (253 mg kg^−1^) and CMCT3 (0.97 mg kg^−1^) had the highest and lowest leaf Cu values, respectively. In addition, Kabata-Pendias [67] defined the phytotoxic range of Cu in plants as 20−100 mg kg^−1^ and based on this limit, leaf and stem Cu concentrations were below the prescribed level in CMC, SMC and SS treatments, while these values reached the phytotoxic range in most of the WB and AFS treatments.

### 2.5. Phytoremediation Efficiency of A. fruticosa

**Figure 3:**
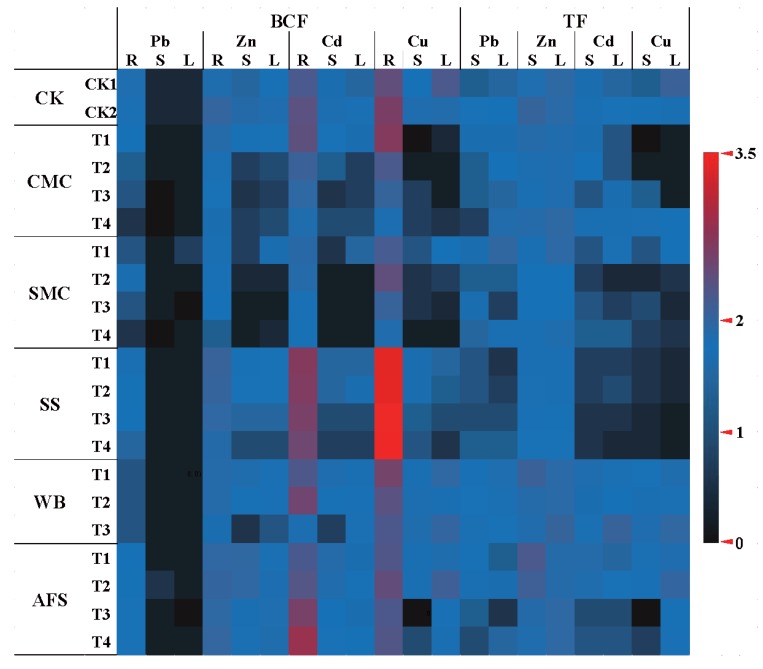
Bioconcentration factors and translocation factors of Pb, Zn, Cd and Cu in *A. fruticosa* tissues grown in treated and untreated tailings. CK1 = Only tailings and CK2 = Tailings plus TSP. The amendment treatments are denoted as T1, T2, T3 and T4. CK1: Control 1; CK2: Control 2; CMC: Cattle manure compost; SMC: Spent mushroom compost; SS: Sewage sludge; WB: Wood biochar; AFS: Agricultural field soil; BCF: Bioconcentration factor; TF: Translocation factor; L: Leaf; S: Stem; R: Root. Plant did not grow in WBT4. (For interpretation of the references to color in this figure legend, the reader is referred to the web version of this article).

Bioconcentration factor (BCF) and translocation factor (TF) values were calculated to evaluate phytoremediation potential of *A. fruticosa* in studied Pb-Zn mine tailings. The plant species showing BCF and TF > 1 should be recommended for phytoextraction, while plants with both BCF and TF < 1 could be suitable for phytostabilization [73]. The BCFs and TFs of Pb, Zn, Cd and Cu varied among the tissues of tested plant and most of the BCFs and TFs of concerned metals in the treatments were lower than 1 (Figure 3). BCFs of Pb in root in all amended samples were significantly lower than both CK1 and CK2, with the minimum being in SMCT4. The lowest Pb BCF in stem and leaf were reported in CMCT4 (0.001) and SMCT3 (0.002), respectively. The TFs of Pb in leaf and stem in WB treatments were higher than other amendments. The possible reason of the higher uptake and translocation of Pb in WB treatments might be the high salinity (Table 1) since the elevated salinity increases metal concentration in soil solution due to the production of soluble inorganic complexes [74]. However, all the BCF and TF values of Pb were lower than 1. The BCF and TF values of Zn in plant tissues were higher than that of Pb, but all values were below 1 except TF of Zn in AFST1 stem. CMC and SMC treated samples showed lower BCFs of Zn in root than the treatments with other amendments. SMCT4 and SST1 were reported to have the minimum (0.07) and maximum (0.7) BCF of Zn in root, respectively. Both the BCF of Zn in stem and leaf were the lowest in SMCT3. The highest TF of Zn was determined in stem of AFST1 (1.1), while the lowest TF was also noticed in the stem of SMCT2 (0.1).

Therefore, our results are similar with Seo et al. [22], who also identified *A. fruticosa* as a metal excluder, with higher Zn, Pb and Cu uptake in roots as well as its limited translocation to above-ground parts. Apart from Pb and Zn, BCFs of Cd in root were more than 1 in SS, WB and AFS treatments. Higher root accumulation of Cd in these treatments might be due to the higher EC values, which increased Cd phytoavailability. Moreover, Sharma and Dubey [75] revealed the mechanism of metal retention in root tissues through the binding of metals to negatively charged cell walls and also through extracellular stabilization that retard its mobility via apoplast. This is the natural strategy of metal excluder plants to restrict transport of any non-essential metals like Cd to above-ground tissues Unlike other metals, Cu uptake in root was higher, presenting BCFs >1 in most of the treatments except in some CMC and SMC treatments. These elevated BCFs of Cu in roots might be because of higher Cu phytoavailability as well as strong binding of Cu to roots, thereby limited transportation through xylem or reverse flow of Cu in the phloem return to roots [76]. But Cu translocation to shoots was lower showing BCFS and TFs in stem and leaf below 1. However, the highest and lowest BCF values of Cu were noticed in roots of SST3 (3.5) and in stem of AFST3 (0.001), respectively; whereas the maximum and minimum TF of Cu were determined in leaf of CK1 (0.8) and in stem of AFST3 (0.001), respectively. Although varied BCF and TF values were reported in different treatments regarding the amendments, the addition of amendments not only decreased metal accumulation in plant tissues but also reduced the TF values, implying the capacity of amendments to improve the phytostabilization efficiency of tested plant. In agreement with the findings of Seo, et al. [22], in our experiment, all metals retained predominantly in plant roots as well as BCF and TF values in plant tissues mostly were below 1, indicating *A. fruticosa* as a potential candidate for phytostabilization of correspondent metals in Pb-Zn mine tailings.

### 2.6. Effect of Amendments on Antioxidant Enzyme Activities and Soluble Protein Content of A. fruticosa Leaf

**Figure 4:**
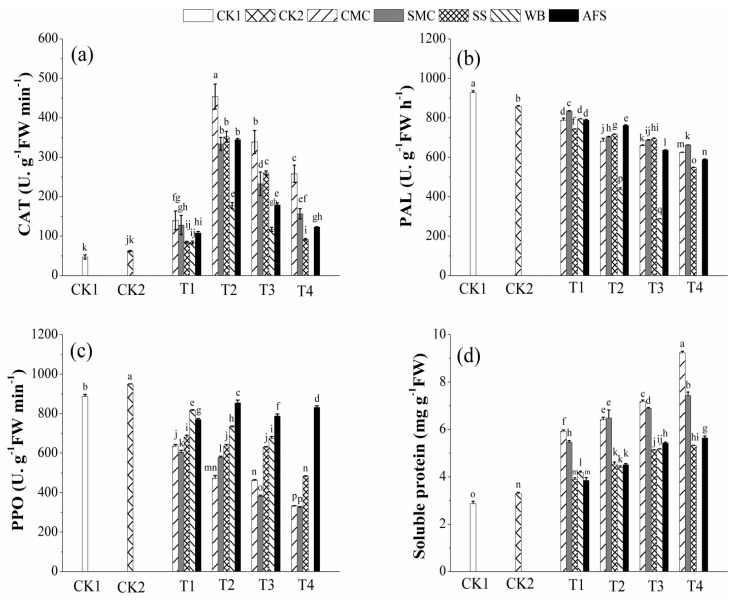
Effect of amendments on CAT (**a**), PAL (**b**), PPO (**c**) activities and soluble protein content (**d**) of *A. fruticosa* leaf. The amendment treatments are denoted as T1, T2, T3 and T4. Error bars represent the standard deviation of the mean (*n* = 3). Letters above the bars refer to the difference at significance level (*p* < 0.05, LSD) among different treatments. CK1: Control 1; CK2: Control 2; CMC: Cattle manure compost; SMC: Spent mushroom compost; SS: Sewage sludge; WB: Wood biochar; AFS: Agricultural field soil. Plant did not grow in WBT4.

To figure out the role of applied amendments in attenuation of metal-induced oxidative injury to plants, the activities of some key antioxidant enzymes such as CAT, PAL and PPO were measured (Figure 4). CAT is a ubiquitous metalloenzyme which is involved in lessening the ROS level by quenching redundant H_2_O_2_ [77,78] and this tetramericheme-containing enzyme has been widely used as a potential bioindicator for metal phytotoxicity [79,80,81]. As revealed by Figure 4a, compared to both CK1 and CK2, CAT activity augmented in all amendment treatments by varying degrees and irrespective of the amendments’ application rates. Interestingly, CK1 (no amendment) showed the lowest CAT activity followed by CK2 (only TSP) and in each amendment treatment, a down-regulated CAT activity from T2-T4 was observed whereas T1 in all cases had the minimum CAT value, indicating inhibition of CAT enzyme in treatments having higher bioavailable metal concentrations (Figure 1). The highest CAT activity was determined in CMCT2 (453.93 U. g^−1^ FW min^−1^), which was 9.65 and 7.28 times higher than CK1 and CK2, respectively. In both CK1 and CK2, CAT enzyme inhibition occurred to a great extent and comparing to the controls, plants grown in amended treatments especially with higher amendment gradients could activate CAT mechanism to counteract metal induced excess H_2_O_2_. Though antioxidant enzyme activities in plant cells supposed to be enhanced under metal stress as a defensive mechanism to combat oxidative stress, many studies reported the declined CAT activity in plant tissues under metal stress due to metal concentrations beyond physiological tolerance level [81,82,83]. In a recent study, Zhang and Ji [84] observed decreased CAT activity in *Orychophragmus violaceus*, *Festuca arundinacea* and *Medicago sativa* under higher stress of metals, which is in support to our findings. Contrarily, Pang et al. [85] reported increased CAT level in leaves of *V. zizanioides* grown in mine tailings with high metal concentrations. Scientists revealed that CAT inhibition occurred when the metal stress in plants exceeded the physiologically controllable range [78,83]. Moreover, since the effects of any single metal and multi-metals on plant antioxidant enzymes are quite different, enzyme activities may not always be corresponded to the concentration of a specific metal [86]. In our study, the observed CAT inhibition might be ascribed to the enzyme inactivation stimulated by ROS, impairment in enzyme synthesis, or alteration in the enzyme subunits’ assemblage [87] as well as metal binding to the active sites of enzyme [88].

PAL acts as the key enzyme in the phenylpropanoid pathway to produce various phenolic derivatives like phenols, flavonoids and lignin [89,90]. PAL is also considered as a potential indicator for physiological stress induced by HMs in plants [80,90,91]. PAL activity showed a clear down-regulation with increased rate of additive applications (Figure 4b), indicating increasing trend in PAL with the increasing bioavailable metal concentrations in amended treatments (Figure 1). Therefore, PAL was effectively induced in plant cells to prevent the ROS induced damage. However, the highest PAL was quantified in the plants of CK1 (927.31 U. g^−1^ FW h^−1^) followed by CK2 (857.56 U. g^−1^ FW h^−1^), which was reasonable to the high phytoavailable metal concentrations in respective tailings samples (Figure 1), and a significant reduction in PAL value was noticed in all the amended treatments in relation to both CK1 and CK2 (Figure 4b). PAL activity ranged from 624.59−788.30 U. g^−1^ FW h^−1^ in CMC, 662.37–833.09 U. g^−1^ FW h^−1^ in SMC, 546.52−742.02 U. g^−1^ FW h^−1^ in SS, 287.11−794.28 U. g^−1^ FW h^−1^ in WB and 588.70−787.67 U. g^−1^ FW h^−1^ in AFS. Increased PAL activity in elevated metal stress might provide active anti-oxidative protection against oxidative injury through regulation of secondary metabolite synthesis. Numerous previous studies also confirmed enhanced PAL activity in plants under HMs stress [80,91,92], which are in agreement with our present observation.

PPO is involved in the oxidation of phenols to quinones and has been widely used in previous studies to evaluate the defensive capacity of plants to resist HMs stress [80,91,92]. A significant difference in PPO activity was noticed between CK1 and CK2, and PPO levels in CMC, SMC, SS and WB treatments significantly reduced with the increasing additive rates as compared to both CK1 and CK2, whereas AFS treatments showed significant declination than the controls irrespective of the additive doses (Figure 4c). Therefore, PPO activities in treatments indicated that amendments not only alleviated bioavailable concentrations of metals (Figure 1) but also efficiently activated PPO enzyme to provide necessary protection under metal stress. The maximum and minimum PPO values were noted in CK2 (949.60 U. g^−1^ FW min^−1^) and SMCT4 (324 U. g^−1^ FW min^−1^). The resulted PPO values were the reflection of multi-metals present in our studied tailings, but PPO activity is mainly regulated by Zn concentrations in plant tissues [93]. In this context, leaf Zn concentrations in different treatments (Figure 2) were in a good correspondence with their respective PPO values. In agreement with our findings, Michael and Krishnaswamy [94], reported a significantly enhanced PPO activity in leaves having high Zn concentration, whereas in Zn deficient leaves PPO reduced slightly. However, in literature, there have been healthy evidences on stimulated PPO activity in plants under elevated HMs concentrations [80,91,92].

Like other abiotic stresses, HMs also exert negative impacts on soluble protein content in plants [79,95,96]. At elevated concentrations, HMs impair the conformation of soluble protein not only by interrupting the folding process for synthesis of new protein, but also tempting the mis-folding of the existing proteins [97,98]. Figure 4d presents the soluble protein contents in plant under different amendments. CK1 and CK2 were significantly different and all the amendment treatments caused significant increase in soluble protein contents over both CK1 and CK2. Compared to CK1, soluble protein content increased by 106.7−222.1% in CMC, 90.3–158.8% in SMC, 35.7–85% in SS, 46–79.9% in WB and 34.2–96.6% in AFS treatments with the increase in amendments’ application rates, indicating a negative correlation with their respective bioavailable metal contents. CMC followed by SMC amendments could pose better amassing of soluble protein in plants than other amendments, showing the highest soluble protein content in CMCT4 (9.24 mg g^−1^ FW), whereas CK1 was the lowest (2.87 mg g^−1^ FW) owing to having the maximum bioavailable metal load (Figure 1). The decrease in soluble protein contents with the increase in HMs’ concentrations in treatments might be due to inadequacy of necessary amino acids and the denaturation of enzymes required for amino acid and protein synthesis [99]. Moreover, the increase in soluble protein content may be attributed to micronutrients (Fe and Mn) addition through applied organic amendments that promote protein synthesis [100,101]. Mehmood et al. [79] also reported increased soluble protein contents in amended metal polluted soils due to reduced bioavailable metal contents, which are corroborated to our findings.

### 2.7. Relationship among Tailings Properties, Plant Growth Parameters, Metal Concentrations in Plant Tissues, Plant Antioxidant Enzymes and Soluble Protein Content

**Figure 5:**
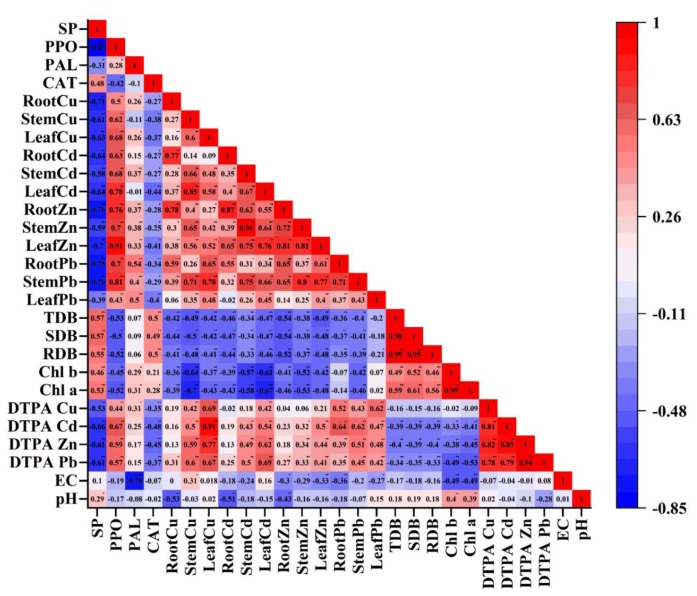
Pearson’s correlation test among tailings’ physicochemical properties, *A. fruticosa* growth, tissue metal concentrations and antioxidant enzyme activitiesafter harvesting.CK1: Control 1; CK2: Control 2; CMC: Cattle manure compost; SMC: Spent mushroom compost; SS: Sewage sludge; WB: Wood biochar; AFS: Agricultural field soil; EC: Electrical conductivity; DTPA: Diethylenetriaminepentaacetic acid; Chl: Chlorophyll; RDB: Root dry biomass; SDB: Shoot dry biomass; TDB: Total dry biomass; CAT: Catalase; PAL: Phenylalanine ammonialyase; PPO: Polyphenol oxidase; SP: Soluble protein(for interpretation of the references to color in this figure legend, the reader is referred to the web version of this article).

The Pearson’s correlation analysis demonstrated that tailings pH had a negative correlation with DTPA metal concentrations (except Cu) and positive correlation with leaf chlorophyll (a and b) content, root and shoot biomass yield (Figure 5). In contrast, EC was negatively correlated with DTPA metal concentrations (except Pb), chlorophyll (a and b) content, root and shoot dry biomass.

Only DTPA Cu content showed a negative correlation with root Zn content; except this, other bioavailable metal contents (Pb, Zn and Cd) were reported to have positive correlation with metal concentrations in plant tissues. Chlorophyll (a and b) content showed a significant negative correlation with leaf metal concentrations (except leaf Pb) whereas root and shoot biomass yield had negative correlation with all the studied metals in plant tissues. A significant (*p* < 0.01) positive correlation was observed between chlorophyll (a and b) content and dry biomass (root and shoot) yield. CAT and soluble protein content were negatively correlated to both bioavailable and leaf metal concentrations (Pb, Zn, Cd and Cu), whereas PAL and PPO had positive correlation with bioavailable and leaf metal concentrations (Figure 5). Except PPO, other antioxidant enzymes (CAT and PAL) and soluble protein content maintained a positive correlation with chlorophyll (a and b) content and root and shoot dry biomass.

## 3. Materials and Methods

### 3.1. Sampling and Characterization of the Studied Pb-Zn Mine Tailings and Amendments

Pb-Zn mine tailings were collected from a tailing pond operated by Shaanxi Qiantongshan Mining Co. Ltd., located in the Qiandongshan Pb and Zn region of Fengxian County (106°24’54”E—107°7’30”E, 33°34’57”N—34°18’21”N). The Qiandongshan Pb and Zn ore reserve is the largest and one of the most typical five national nonferrous metal planning mines that accounts for 25.3% of the total reserves of Fengxian County [102].

Mine wastewater, atmospheric deposition and mine tailings are the predominant pollution sources in this region. Mine tailings were sampled randomly using shovels at a depth of 0−20 cm. A total of 300 samples were collected from different points covering the whole tailing pond and transferred to the laboratory using plastic bags. Collected tailings were homogenized, air-dried in room temperature, manually crushed, thoroughly mixed and then passed through 2 mm sieve for further experiment and physicochemical analyses. General physicochemical properties and HMs content of studied tailings and amendments have been illustrated in Table 2.

Among the studied amendments, AFS was collected from a field of Fengxian County (Shaanxi, China). Other amendments viz. CMC, SMC, WB (derived from apple wood and pyrolyzed at 400 °C), SS, TSP (46% P_2_O_5_) were purchased from the local farms and producers of Yangling District (Shaanxi, China). pH and EC of tailings and amendments were measured (1:5 H_2_O *w*/*v*) using a pH meter (Mettler Toledo 320-S, Shanghai Bante Instrument Co., Ltd., Shanghai, China) and a EC meter (DDS-307, Shanghai Bante Instrument Co. Ltd., Shanghai, China) according to USEPA Method 9045D [103] and ASTM D1125-95 [104], respectively. Water holding capacity (WHC) of air dried tailings and AFS was examined following the method used by Mahar et al. [44]. Particle size of sand, silt and clay was measured using a Mastersizer 2000E laser difractometer (Malvern, Worcestershire, UK) as described by Sochan et al. [105]. CEC was evaluated according to USEPA Method 9080 [106]. Total carbon (%) was measured by a CN Analyzer (Vario Max, Elementar, Langenselbold, Germany). Total nitrogen (TN) content was determined by Kjeldahl method. Total P (TP) and total K (TK) contents were analyzed according to molybdenum-antimony colorimetry and flame photometry methods, respectively [107]. Available N was determined using a continuous flow analyzer (Bran and Luebbe AA3, Norderstedt, Germany) according to the method followed by Alvarenga et al. [108]. Available P was extracted with 0.5 M NaHCO_3_ (pH 8.5) and the amount in the extract was measured by blue coloration method using UV-vis spectrophotometer (Model UV-2450, Shimadzu, Kyoto, Japan) [109]. Available K was determined using flame photometer (FP 6410, Shanghai Bante Instrument Co. Ltd.) after extraction with 1 M NH_4_OAc (pH 7.0) [110]. Total heavy metal concentrations were analyzed according to USEPA Method 3051A [111] with minor modifications by digesting 0.2 g sample with 15 mL of tri-acidic mixture (HNO_3_: HCl: HClO_4_ = 1:3:1). The bioavailable metal fraction of Pb, Zn, Cd and Cu were extracted with diethylenetriamine penta-acetic acid (DTPA) as outlined by Lindsay and Norvell [112]. The metal concentrations were determined using atomic absorption spectrophotometer (AAS Z-2000, Hitachi, Kawaguchi, Japan).

The studied tailings were considered as sandy loam (USDA classification) containing 66.78% sand, 21.73% silt and 11.5% clay. The tailings were neutral in reaction (pH 7.33), with moderate EC (1.27 dS m^−1^), low CEC and WHC (10.53 cmol kg^−1^ and 15.36%, respectively) and poor nutrient contents (N, P and K). Like other Pb-Zn mine tailings [113,114], concentrations of total Pb, Zn, Cd and Cu in our studied tailings were high, namely 11.23, 30.88, 63 and 1.06 times higher than the environmental quality standard for soils suitable for the growth of crops and forest trees in China (GB15618-1995, Grade III). DTPA-extracted concentrations of Pb, Zn, Cd and Cu in tailings were quantified as 296, 1325, 9.17 and 126 mg kg^−1^, respectively.

### 3.2. Pot Experiment

The pot experiment was conducted at a greenhouse of Northwest A & F University (Yangling (34°15’60”N, 108°3’46”E), Shaanxi, China). CMC, SMC, SS, WB and AFS were applied to tailings at 5%, 10%, 20% and 30% *w*/*w* ratio, 5%, 10% and 20%, 30% *w*/*w* ratio, 2.5%, 5%, 10% and 20% *w*/*w* ratio, 2.5%, 5%, 10% and 20% *w*/*w* ratio, and 5%, 10%, 20% and 30% *w*/*w* ratio, respectively. Hereafter, the amendment treatments are denoted as T1, T2, T3 and T4 for each ratio of five amendments. TSP was applied to each treatment at a P/Pb molar ratio of 4:1. Pb content in amendments was not considered for TSP amount calculation. Seven kg of air-dried tailings and TSP (at P: Pb = 4:1) were mixed thoroughly with the amendments in desired ratios and then placed into plastic pots (28 × 19 × 21 cm). There were three replicates for each treatment. Un-amended tailings were kept as control 1(CK1) and tailings plus TSP (at P: Pb = 4:1) was considered as control 2 (CK2) with triplicate. The prepared mixtures were incubated for 30 days to equilibrate prior to sowing. Meanwhile, the moisture was maintained at 70% of their water holding capacity with tap water. All the pots were arranged in a randomized block design in greenhouse. 20 seeds were sown in each pot and after 2 months, the seedlings were thinned to 5 per pot. The plants were harvested 180 days after seeding.

### 3.3. Sample Analyses

After harvesting of plants, approximately 300 g tailings sample were collected from each pot and then naturally air-dried, ground to pass through a 2 mm nylon sieve and analyzed for pH, EC and DTPA metal concentrations (Pb, Zn, Cd and Cu) according to the methods mentioned earlier.

At harvest, the aboveground parts (stems and leaves) and underground parts (roots) of *A. fruticosa* plants were removed from pots and placed into plastic bags separately. The plant parts were carefully washed with running tap water followed by deionized water, oven dried to constant weight at 70 °C and dry biomass of shoot and root were determined. Dried plant samples were crushed into fine powder by a stainless-steel grinder (FW-100, Shanghai Bante Instrument Co. Ltd., Shanghai, China), passed through a 100 mesh nylon sieve (0.15 mm) and stored in plastic bags. For analysis of HMs, powdered plant samples were digested with concentrated HNO_3_-HClO_4_ (4:1) [115]. The resultant solutions were analyzed for total concentration of Pb, Zn, Cd and Cu using AAS (Hitachi FAAS Z-2000). Quantification of chlorophylls (chlorophyll a and chlorophyll b) was done following the method used by Ali et al. [91]. Fresh leaf samples (0.2 g) from each treatment were grinded and extracted with 80% acetone for 24 h in dark and measured the absorbance at 663 and 645 nm for the chlorophyll a and chlorophyll b contents, respectively. CAT activity of plant leaf was detected according to the method adopted by Cheng et al. [77]. PAL and PPO were determined following the standard method used by Ahammed et al. [116]. Soluble protein content was quantified by the method of Bradford [117].

### 3.4. Bioconcentration Factor and Translocation Factor

To measure the effect of amendments on HMs uptake and translocation in *A. fruticosa* tissues, the BCF and TF were calculated. The BCF means the ability of a plant species to uptake a metal into its root tissues from soil. The TF refers to the capacity of a plant to translocate the metal from its root to aerial parts [118,119,120]. The BCF and TF were calculated as follows:

BCF = Metal concentration in plant tissue/metal concentration in soil (1)

TF = Metal concentration in aerial tissue/Metal concentration in root (2)

### 3.5. Quality Control and Statistical Analyses

Several steps for quality control and assurance were maintained during sample preparation and chemical analysis, including sample triplicates, method blanks, the use of certified reference materials for instrumental calibration and detection limit verification. The certified reference materials for soil (GBW07403) and plants (GBW07603, bush twigs and leaves) were obtained from the National Research Center for Certified Reference Materials (Beijing, China). The recovery of HMs varied within the acceptable limits of 94.3 to 108.4%.

During the entire analyses, all of the results were calculated from the triplicate of the analytical data and average readings have been presented with standard deviation. All experimental data were subjected to a one-way analysis of variance (ANOVA) performing Tukey’s LSD (Least significant difference) test at a significant level of *p* < 0.05 using SPSS 22 software (SPSS Inc., Chicago, IL, USA). Simple correlations among the variables were estimated with Pearson’s test.In addition, all the graphs were prepared using OriginPro 9.0 software (Originlab Corporation, Northampton, MA, USA).

## 4. Conclusions

In the current study, five organic amendments combined with an inorganic phosphate (TSP) were tested to reduce metal solubility and phytotoxicity in *A. fruticosa*. The bioavailable Pb, Zn, Cd and Cu concentrations of studied tailings were potentially reduced by mixing of amendments except in WB treatments. However, plant growth significantly improved in CMC and SMC treatments. *A. fruticosa* had higher concentrations of Pb, Zn, Cd and Cu in roots, but translocation to aerial parts was low, indicating its’ strong phytostabilization potential of metal polluted sites. Application of amendments decreased bioavailable metal concentrations of tailings and subsequently reduced PAL and PPO activities in plant leaves, whereas inhibition of CAT activity was observed in CK1, CK2 and T1 of each amendment due to having metal load beyond plants’ physiological tolerance level and from T2-T4 CAT declined with the increased amendment rates. Soluble protein content increased gradually in all amendment treatments with the additive induced reduction in bioavailable metal concentrations in the treated tailings. Therefore, our results demonstrated that the combinations of organic and inorganic amendments were effective for stabilizing metals of concern and thereby could support vegetation coverage in Pb-Zn mine tailings reducing phytotoxicity of metals. Further research is required to investigate the long-term effects of these amendments and *A. fruticosa* under field conditions which would provide deeper understanding of the ecotoxicological findings observed.

## Figures and Tables

**Table 1:** Effect of different amendments on pH and EC of treated and untreated tailings samples; and chlorophyll (Chl) contents and plant dry biomass (mean ± standard deviation; *n* = 3) of *A. fruticosa* grown in treated and untreated tailings samples.

Treatment	pH	EC (dS m^−1^)	Chl a Content (mg g^−1^FW)	Chl b Content (mg g^−1^FW)	Shoot Dry Biomass (g pot^−1^)	Root Dry Biomass (g pot^−1^)	Total Dry Biomass (g pot^−1^)
CK1	6.64 ± 0.14 ^d–g^	1.08 ± 0.03 ^j^	1.70 ± 0.01 ^h^	0.68 ± 0.00 ^g^	1.80 ± 0.00 ^e^	0.72 ± 0.00 ^f^	2.52 ± 0.00 ^e^
CK2	6.63 ± 0.25 ^d–h^	1.11 ± 0.04 ^ij^	0.98 ± 0.07 ^k^	0.37 ± 0.03 ^j^	1.78 ± 0.01 ^e^	0.71 ± 0.00 ^f^	2.49 ± 0.01 ^e^
CMCT1	6.69 ± 0.09 ^c–f^	1.24 ± 0.07 ^h–j^	2.22 ± 0.03 ^f^	0.71 ± 0.00 ^g^	11.82 ± 0.15 ^a^	16.58 ± 3.44 ^a^	28.40 ± 3.59 ^a^
CMCT2	6.56 ± 0.19 ^e–i^	1.37 ± 0.11 ^f–j^	2.36 ± 0.01 ^e^	0.76 ± 0.00 ^f^	12.21 ± 4.60 ^a^	19.32 ± 4.29 ^a^	31.53 ± 5.58 ^a^
CMCT3	6.63 ± 0.05 ^d–h^	1.59 ± 0.17 ^e–g^	2.47 ± 0.01 ^d^	0.75 ± 0.00 ^f^	13.48 ± 1.43 ^a^	19.45 ± 1.88 ^a^	32.93 ± 1.45 ^a^
CMCT4	6.62 ± 0.13 ^d–h^	1.79 ± 0.34 ^de^	1.71 ± 0.01 ^h^	0.57 ± 0.00 ^h^	5.15 ± 2.26 ^cd^	5.55 ± 3.99 ^cd^	10.70 ± 6.23 ^cd^
SMCT1	6.85 ± 0.11 ^b–d^	1.08 ± 0.03 ^j^	3.08 ± 0.02 ^b^	1.13 ± 0.01 ^b^	5.25 ± 1.58 ^cd^	4.40 ± 2.25 ^de^	9.65 ± 3.83 ^d^
SMCT2	6.84 ± 0.17 ^b–d^	1.12 ± 0.06 ^ij^	3.28 ± 0.03 ^a^	1.16 ± 0.02 ^a^	7.90 ± 1.88 ^bc^	8.43 ± 1.44 ^bc^	16.33 ± 3.21 ^bc^
SMCT3	6.69 ± 0.15 ^c–f^	1.28 ± 0.06 ^g–j^	3.29 ± 0.00 ^a^	1.17 ± 0.00 ^a^	8.17 ± 3.90 ^b^	9.48 ± 5.38 ^b^	17.65 ± 9.27 ^b^
SMCT4	7.06 ± 0.10 ^b^	1.41 ± 0.01 ^f–i^	2.91 ± 0.02 ^c^	0.99 ± 0.01 ^c^	5.22 ± 2.82 ^cd^	6.02 ± 4.51 ^b–d^	11.24 ± 7.32 ^cd^
SST1	6.36 ± 0.15 ^h–j^	1.24 ± 0.02 ^h–j^	2.16 ± 0.02 ^g^	0.83 ± 0.01 ^e^	1.87 ± 0.00 ^e^	0.74 ± 0.00 ^ef^	2.61 ± 0.00 ^e^
SST2	6.39 ± 0.21 ^g–j^	1.52 ± 0.07 ^e–h^	1.26 ± 0.02 ^i^	0.47 ± 0.00 ^i^	1.85 ± 0.00 ^e^	0.73 ± 0.00 ^f^	2.58 ± 0.00 ^e^
SST3	6.44 ± 0.10 ^f–j^	1.98 ± 0.12 ^d^	1.03 ± 0.01 ^j^	0.30 ± 0.01 ^k^	1.80 ± 0.00 ^e^	0.72 ± 0.00 ^f^	2.52 ± 0.00 ^e^
SST4	6.25 ± 0.13 ^j^	2.31 ± 0.05 ^c^	0.63 ± 0.02 ^m^	0.20 ± 0.06 ^l^	1.75 ± 0.00 ^e^	0.71 ± 0.00 ^f^	2.46 ± 0.00 ^e^
WBT1	6.32 ± 0.20 ^ij^	1.61 ± 0.25 ^ef^	0.39 ± 0.00 ^n^	0.13 ± 0.00 ^no^	2.52 ± 0.00 ^de^	0.85 ± 0.00 ^ef^	3.37 ± 0.00 ^e^
WBT2	6.49 ± 0.19 ^f-j^	1.92 ± 0.15 ^d^	0.17 ± 0.05 ^o^	0.17 ± 0.01 ^m^	1.80 ± 0.00 ^e^	0.78 ± 0.00 ^ef^	2.57 ± 0.00 ^e^
WBT3	6.92 ± 0.14 ^bc^	3.56 ± 0.67 ^b^	0.10 ± 0.00 ^o^	0.10 ± 0.01 ^o^	1.76 ± 0.00 ^e^	0.75 ± 0.00 ^ef^	2.51 ± 0.00 ^e^
WBT4	7.35 ± 0.11 ^a^	4.65 ± 0.26 ^a^	-	-	-	-	-
AFST1	6.68 ± 0.21 ^c–f^	1.18 ± 0.06 ^ij^	0.38 ± 0.02 ^n^	0.14 ± 0.01 ^n^	1.87 ± 0.01 ^e^	0.80 ± 0.00 ^ef^	2.67 ± 0.01 ^e^
AFST2	6.59 ± 0.16 ^d–i^	1.19 ± 0.01 ^ij^	0.74 ± 0.01 ^l^	0.28 ± 0.00 ^k^	2.22 ± 0.30 ^e^	0.86 ± 0.04 ^ef^	3.09 ± 0.34 ^e^
AFST3	6.44 ± 0.00 ^f–j^	1.19 ± 0.01 ^ij^	1.02 ± 0.01 ^j^	0.39 ± 0.00 ^j^	2.54 ± 0.15 ^de^	0.89 ± 0.02 ^ef^	3.44 ± 0.15 ^e^
AFST4	6.83 ± 0.06 ^b–e^	1.20 ± 0.01 ^ij^	2.23 ± 0.02 ^f^	0.86 ± 0.01 ^d^	2.58 ± 0.13 ^de^	0.91 ± 0.00 ^ef^	3.50 ± 0.13 ^e^

Means followed by the different letter(s) within each column are significantly different at *p* < 0.05 according to LSD test. CK1: Control 1; CK2: Control 2; CMC: Cattle manure compost; SMC: Spent mushroom compost; SS: Sewage sludge; WB: Wood biochar; AFS: Agricultural field soil; Chl: Chlorophyll; EC: Electrical conductivity. Plant did not grow in WBT4.

**Table 2:** Basic physicochemical properties of the studied Pb-Zn mine tailings and amendments (mean ± standard deviation; *n* = 3).

Parameters	Tailings	AFS	CMC	SMC	SS	WB	TSP
pH (1:5 H_2_O)	7.33 ± 0.07	7.85 ± 0.07	7.75 ± 0.04	7.36 ± 0.04	5.44 ± 0.02	10.50 ± 0.03	2.52 ± 0.01
EC (1:5) (dS m^−1^)	1.27 ± 0.01	0.15 ± 0.01	5.84 ± 0.01	5.87 ± 0.08	2.41 ± 0.02	26 ± 0.4	7.54 ± 0.1
Clay%	11.5	36.96	-	-	-	-	-
Silt%	21.7	53.45	-	-	-	-	-
Sand%	66.8	9.59	-	-	-	-	-
Textural class	Sandy loam	Silty clay loam	-	-	-	-	-
CEC (cmol kg^−1^)	10.53 ± 0.2	33.94 ± 0.9	62.41 ± 0.8	269 ± 0.7	72.60 ± 0.6	143 ± 0.3	127 ± 0.9
Water holding capacity (%)	15.36 ± 0.4	23.40 ± 0.2	-	-	-	-	-
Moisture content (%)	0.37 ± 0.01	0.92 ± 0.09	7.27 ± 0.2	8.6 ± 0.4	8.82 ± 0.1	12.46 ± 0.5	-
C%	4.40 ± 0.0	1.30 ± 0.0	30.49 ± 0.0	25.31 ± 0.0	32.28 ± 0.1	41.50 ± 0.1	-
Total N (g kg^−1^)	0.44 ± 0.0	0.33 ± 0.0	13.04 ± 0.1	11.44 ± 0.1	21.30 ± 0.1	9.50 ± 0.0	-
Total P (g kg^−1^)	0.20 ± 0.0	5.62 ± 0.0	8.75 ± 0.0	1.54 ± 0.0	2.19 ± 0.0	3.88 ± 0.0	-
Total K (g kg^−1^)	8.49 ± 0.0	10.87 ± 0.0	11.29 ± 0.0	8.58 ± 0.0	12.31 ± 0.0	82.50 ± 0.0	-
Available N (mg kg^−1^)	3.77 ± 0.1	-	-	-	-	-	-
Available P (mg kg^−1^)	6.69 ± 0.1	9.04 ± 0.1	351 ± 1.0	154 ± 0.1	529 ± 0.7	146 ± 0.8	-
Available K (mg kg^−1^)	3.98 ± 0.1	26.51 ± 0.1	197 ± 0.4	86.23 ± 0.	498 ± 0.2	1051 ± 0.4	-
Total Pb (mg kg^−1^)	5614 ± 51	62.65 ± 5.9	21.42 ± 9.7	60.06 ± 3.6	38.34 ± 4.3	69.31 ± 2.9	-
Total Zn (mg kg^−1^)	15439 ± 114	109 ± 2.9	263 ± 2.4	90.84 ± 6.6	411 ± 7.1	64.35 ± 3.2	-
Total Cd (mg kg^−1^)	63.58 ± 1.1	2.73 ± 0.2	1.87 ± 0.2	4.00 ± 0.1	1.54 ± 0.1	4.57 ± 0.5	-
Total Cu (mg kg^−1^)	425 ± 2.9	19.67 ± 1.1	37.40 ± 0.3	14.93 ± 0.5	51.46 ± 0.7	25.49 ± 0.5	-
Total Ni (mg kg^−1^)	8.54 ± 1.2	22.49 ± 0.5	2.41 ± 0.1	1.14 ± 0.1	13.48 ± 1.9	3.82 ± 0.2	-
Total Cr (mg kg^−1^)	8.46 ± 0.4	20.35 ± 2.9	0.96 ± 0.1	7.58 ± 1.4	65.30 ± 4.8	39.88 ± 1.9	-
Total Ca (mg kg^−1^)	94,391 ± 2920	25,631 ± 773	19,146 ± 765	81,112 ± 1394	13,246 ± 680	54,197 ±1579	-
Total Mg (mg kg^−1^)	9051 ± 365	7354 ± 206	7286 ± 309	4761 ± 293	4127 ± 46.2	8340 ± 336	-
Total Fe (mg kg^−1^)	41,624 ± 387	25,628 ± 610	5488 ± 192	4920 ± 55.2	8935 ± 26.2	9544 ± 321	-
Total Na (mg kg^−1^)	305 ± 14.8	437 ± 41.6	2603 ± 122	617 ± 10.3	1411 ± 60.8	416 ± 18.6	-
Total Mn (mg kg^−1^)	1532 ± 30.5	630 ± 25.5	230 ± 11	216 ± 8.2	141.80 ± 3.8	414 ± 18.0	-
DTPA Pb (mg kg^−1^)	296 ± 4.5	9.14 ± 0.2	11.58 ± 0.4	6.29 ± 0.7	9.85 ± 0.2	13.39 ± 0.3	-
DTPA Zn (mg kg^−1^)	1325 ± 3.6	4.86 ± 0.2	95 ± 2.6	5.46 ± 0.3	24.12 ± 0.1	1.80 ± 0.2	-
DTPA Cd (mg kg^−1^)	9.17 ± 0.04	0.15 ± 0.0	0.17 ± 0.0	0.13 ± 0.01	0.11 ± 0.01	0.28 ± 0.01	-
DTPA Cu (mg kg^−1^)	126 ± 4.7	1.14 ± 0.03	5.82 ± 0.5	0.95 ± 0.02	3.81 ± 0.3	1.15 ± 0.1	-

Values indicate mean of one sample with three replications. AFS: Agricultural field soil; CMC: Cattle manure compost; SMC: Spent mushroom compost; SS: Sewage sludge; WB: Wood biochar; TSP: Triple superphosphate; EC: Electric conductivity; CEC: Cation exchange capacity; (-): Not measured.
